# Vitamin B_12_ deficiency-induced pseudothrombotic microangiopathy without macrocytosis presenting with acute renal failure: a case report

**DOI:** 10.1186/s13256-018-1815-8

**Published:** 2018-10-03

**Authors:** Jennifer Vanoli, Andrea Carrer, Roberto Martorana, Guido Grassi, Michele Bombelli

**Affiliations:** 1 Divisione di Clinica Medica, Ospedale San Gerardo, Monza, Italy; 2 University of Milano-Bicocca, Milan, Italy; 3 Divisione di Ematologia, Ospedale San Gerardo, Monza, Italy; 4 Istituto di Ricerca a Carattere Scientifico IRCCS Multimedica, Sesto San Giovanni, Milan, Italy

**Keywords:** Pseudothrombotic thrombocytopenic purpura, Vitamin B_12_ deficiency, Hemolytic anemia, Thrombocytopenia

## Abstract

**Background:**

Vitamin B_12_ deficiency-induced thrombotic microangiopathy, known as pseudothrombotic microangiopathy, is a rare condition which resembles the clinical features of thrombotic thrombocytopenic purpura but requires a markedly different treatment. Most cases of vitamin B_12_ deficiency have only mild hematological findings, but in approximately 10% of patients life-threatening conditions have been reported.

**Case presentation:**

We report a case of a 46-year-old Moroccan man presenting with severe hemolytic anemia, thrombocytopenia, and renal failure in absence of macrocytosis, thus mimicking a genuine thrombotic thrombocytopenic purpura. Rapid improvement of renal function observed with only hydration and transfusions of packed red blood cells and the presence of pancytopenia suggested a bone marrow deficiency associated to a hemolytic component of unclear origin. Detection of low levels of vitamin B_12_ and rapid *restitutio ad integrum* with its replacement supported the diagnosis of pseudothrombotic thrombocytopenic purpura caused by vitamin B_12_ deficiency.

**Conclusions:**

Diagnosis of pseudothrombotic thrombocytopenic purpura caused by vitamin B_12_ deficiency might be difficult. Awareness of clinicians toward this differential diagnosis might spare patients from unnecessary therapeutic plasma exchange that is burdened by morbidity and mortality.

**Electronic supplementary material:**

The online version of this article (https://doi.org/10.1186/s13256-018-1815-8) contains supplementary material, which is available to authorized users.

## Background

Thrombotic microangiopathies are rare conditions associated with a mortality of 10–20%. They include heterogeneous disorders, such as thrombotic thrombocytopenic purpura and hemolytic uremic syndrome, characterized by microangiopathic hemolytic anemia, severe thrombocytopenia, and organ damage by microvascular occlusion [1].

Vitamin B_12_ deficiency-induced thrombotic microangiopathy, known as pseudothrombotic microangiopathy, is a rare condition which resembles the clinical features of thrombotic thrombocytopenic purpura but requires a markedly different treatment. Most cases of vitamin B_12_ deficiency have only mild hematological findings; however, in approximately 10% of patients, life-threatening conditions have been reported [2].

Here we report a case of pseudothrombotic thrombocytopenic purpura caused by vitamin B_12_ deficiency that is challenging because of the presentation with renal failure in the absence of macrocytosis, thus mimicking a genuine thrombotic thrombocytopenic purpura. Distinguishing between the two allows avoidance of unnecessary aggressive treatments that are burdened by morbidity and mortality.

## Case presentation

A 46-year-old Moroccan man with a history of cocaine and alcohol abuse, former smoker of 10 packs/year, detained in a penitentiary for 3 months, presented to an emergency department because of the finding by penitentiary doctors of severe anemia: hemoglobin (Hb) 43 g/L. He did not report previous concomitant comorbidities and he did not take any medication prior to hospital admission. It was difficult to collect a detailed family history because of a language barrier; he worked as a street vendor. He complained of progressive fatigue, arthromyalgia, upper finger paresthesia, mild abdominal pain, left ear tinnitus, and recurring headache for the previous 2 months. He denied fever, bleeding, and changes in bowel habits. At admission, severe normocytic anemia with Hb of 36 g/L, mean corpuscular volume (MCV) 87 fl, hematocrit (htc) 10.8%, and random distribution of red cell width (RDW) of 27% was confirmed, with neutropenia (0.59 ×  10^9^/L) and normal platelet count (15 × 10^9^/L). On presentation he was oriented, afebrile (axillary temperature of 36 °C), and hemodynamically stable with blood pressure of 110/70 mmHg and a heart rate of 80 per minute. A physical examination showed pale skin, slight epigastralgia, and left tympanic membrane perforation; no lymphadenopathy, purpura, or hepatosplenomegaly were detected. A neurological examination was normal without any motor, sensory, or cranial nerves dysfunction except for slight upper finger paresthesia. Initial laboratory investigations revealed renal impairment with creatinine up to 176.8 μmol/L and azotemia 24.9 mmol/L, marked anisopoikilocytosis and multiple schistocytes (10%) on peripheral smear, lactate dehydrogenase (LDH) increase (19.7 μkat/L), haptoglobin less than 1 mg/L, and normal bilirubinemia (17.1 μmol/L). Coagulation studies were normal except for slight elevation of D-dimer (2.63 nmol/L); markers of inflammation were negative; liver function was normal with aspartate aminotransferase (AST) 30 U/L and alanine aminotransferase (ALT) 18 U/L. A direct Coombs test was negative, reticulocytes count was consistent with inappropriate bone marrow response (reticulocytes production index 0.061), and ferritin was within normal range.

Hydration with normal saline and blood transfusions with packed red blood cells were started. We observed an initial improvement of renal function but anemia did not improve enough despite transfusions with five bags of packed red blood cells, and it was associated to hemolysis (schistocytes, LDH further increase and haptoglobin consumed). In parallel we observed a progressive rapid decrease of platelet count down to 46 × 10^9^/L and severe neutropenia was persistent without peripheral blasts. A diagnosis of thrombotic thrombocytopenic purpura and hemolytic uremic syndrome was considered, but the rapid improvement of renal function with only hydration and the pancytopenia suggested a bone marrow deficiency associated to a hemolytic component of unclear origin. We decided to keep on with blood transfusion support and to strictly monitor our patient until the results of further investigations; meanwhile, we started intramuscular vitamin B_12_ 1000 mcg daily because of extremely low, barely detectable, plasma levels (< 36.9 pmol/L). Serology for cytomegalovirus, Epstein–Barr virus, parvovirus B19, and *Toxoplasma gondii* were negative (past infection) as were serology for human immunodeficiency virus and hepatitis virus. Bone marrow aspirate revealed normal cellularity with different cell types at various stages of maturation and without dysplastic alterations. An abdomen ultrasound showed normal kidneys and very slight splenomegaly (bipolar diameter 12.2 cm) without hepatomegaly and lymphadenopathy. See Additional file 1: Figure S1 for the timeline of the diagnostic and therapeutic flow of the present case report.

**Fig. 1:**
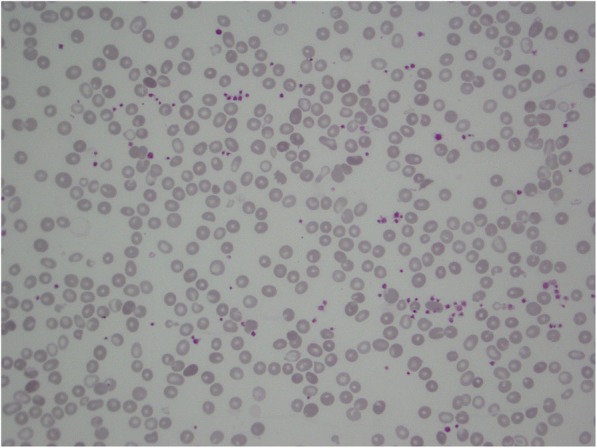
Blood smear obtained a week after the beginning of the treatment showing marked anisopoikilocytosis with few teardrop cells (5%) and disappearance of schistocytes

A week after beginning the vitamin B_12_ supplement we observed a dramatic hematological improvement with simultaneous decrease of hemolysis indexes; marked anisopoikilocytosis with teardrop cells (5%) persisted on peripheral smear without schistocytes (Fig. 1). Anti-parietal cell antibodies were negative; upper endoscopy showed moderate gastric corpus atrophy, without presence of *Helicobacter pylori*. Recovery was complicated by pneumonia and urinary tract infections which were treated with intravenously administered amoxicillin/clavulanic acid.

We discharged our asymptomatic patient 2 weeks after admission with normal renal function, moderate normocytic anemia (85 g/L), and normal platelet and neutrophil count (484 × 10^9^/L and 2.79 × 10^9^/L, respectively).

At a follow-up of 6 months, he was alive but it was not possible to collect further medical information because we were informed that he was a fugitive.

## Discussion and conclusions

Thrombotic microangiopathies syndromes, such as thrombotic thrombocytopenic purpura, are defined by clinical and pathological characteristics. The clinical features include microangiopathic hemolytic anemia, thrombocytopenia, and organ injury while the pathologic characteristic is vascular damage that is manifested by arteriolar and capillary thrombosis [3].

The classic presentation of vitamin B_12_ deficiency consists of macrocytic anemia with or without neurologic manifestations [4], but this condition  may present also with hemolytic anemia, thrombocytopenia, and schistocytosis, mimicking thrombotic microangiopathy in approximately 2.5% of cases [5]. Discriminating between pseudothrombotic microangiopathy and a true microangiopathy hemolytic anemia is of paramount importance, as the treatments are markedly different.

Vitamin B_12_ is essential for deoxyribonucleic acid (DNA) synthesis, hematopoietic cell division, and myelination. It is also needed as a cofactor for two reactions: the first one is the generation of methionine from homocysteine and the second is the conversion of methylmalonyl-coenzyme A to succinyl-coenzyme [6]. Hence, vitamin B_12_ deficiency results in the accumulation of homocysteine and methylmalonic acid. The pathogenesis of vitamin B_12_ deficiency-induced thrombotic microangiopathy is poorly understood but many studies suggest that hyperhomocysteinemia may be involved leading to clot activation and endothelial dysfunction, which results in fragmentation of erythrocytes to schistocytes [7, 8]. Moreover, vitamin B_12_ deficiency increases red blood cell membrane rigidity that results in intramedullary hemolysis and entrapment in the microcirculation [9, 10].

We report the case of a pseudothrombotic microangiopathy due to vitamin B_12_ deficiency, which is a rare manifestation described in only a few case reports in the literature, with the peculiarity of acute kidney failure and the absence of macrocytosis that made differential diagnosis cumbersome.

Although our case is similar to other pseudothrombotic thrombocytopenic purpuras because of inappropriate low reticulocyte count and lower bilirubin levels than expected [11], the finding of leukopenia and especially the presentation with acute renal kidney are unusual. Acute kidney injury and altered mental status, in fact, are more typical of true thrombotic microangiopathy. Only two other case reports described pseudothrombotic thrombocytopenic purpura associated to acute kidney injury, both explained by dehydration and hypoxia [12, 13]. In our case renal impairment was probably due to severe hypoxia, a consequence of anemia, since it rapidly improved after transfusions (Table 1).

**Table 1:** Comparison of four similar unusual cases of pseudothrombotic thrombocytopenic purpura caused by vitamin B_12_ deficiency presenting with renal failure and a lack of macrocytosis

Clinical characteristics	Patients series (Author and reference)				
	Present case report	Kandel *et al*. [12]	Walter *et al*. [13]	Dalsania *et al*. [14]	Garderet *et al*. [15]
Gender/Age	M/46	F/86	F/77	M/48	M/38
Hemoglobin (g/L) [140–180]	36	32	55	50	45
MCV (fl) [80–99]	87	127	120	80.2	90
WBC/neutrophil (× 10^9^/L) [4–11/1.5–7.5]	2.52/0.59	6.5/−	5.9/−	6.3/−	2.2/−
Platelets (× 10^9^/L) [140–440]	46	59	40	38	5
Creatinine (μmol/L) [59–103]	176.8	168	300.6	97.2	Normal
Corrected reticulocyte count/Absolute reticulocyte count (× 10^9^/L) [0.5–2/25–75]	0.18/8.7	1.6/−	1.2/−	0.23/13	−/10
Schistocytosis	Present	Present	Present	Present	Present
LDH (μkat/L) [2.25–3.76]	24.6	118.2	66.5	150.1	323.7
Total/direct bilirubin (μmol/L) [< 24/< 5.1]	17.1/−	–	63.3/17.1	20.5/5.1	205.3/85.5
Haptoglobin (mg/L) [300–2000]	< 1	140	Undetectable	< 100	< 100
Coombs test	Negative	Negative	Negative	Negative	Negative
Serum B_12_ (pmol/L) [145–569]	< 36.9	28	Undetectable	352.7	Undetectable
Methylmalonic acid level (μmol/l) [0–0.4]	Not performed	0.753	Not performed	25,417	Not performed
Neurological symptoms	None	Present	Present	None	None
TPE	NO	NO	YES	YES	YES

*F* female, *LDH* lactate dehydrogenase, *M* male, *MCV* mean corpuscular volume, *TPE* therapeutic plasma exchange, *WBC* white blood cells

The absence of macrocytosis further complicated the diagnosis because it is a common feature of vitamin B_12_ deficiency. However, there are two other cases of pseudothrombotic thrombocytopenic purpura in the literature that reported mean corpuscular volume in normal range [14, 15]. This could possibly be explained by the presence of abundant schistocytes as the small size of schistocytes decreases the mean corpuscular volume and increases the red cell distribution width. Another plausible hypothesis that has been considered, after having excluded an iron deficiency, was an underlying microcytic hemoglobinopathy, since our patient comes from North Africa where this is endemic. Unfortunately, this could not be confirmed due to lack of a complete blood count prior to the admission and at follow-up to obtain an Hb electrophoresis (Table 1). Of interest, LDH levels were lower than expected from similar cases in the literature [11], resembling more what is observed in thrombotic thrombocytopenic purpura.

In conclusion, this was a challenging case of pseudothrombotic thrombocytopenic purpura caused by vitamin B_12_ deficiency because the presentation with renal failure and the lack of macrocytosis and LDH elevation mimicked a genuine thrombotic thrombocytopenic purpura. Inadequate bone marrow response to hemolysis coupled with low white blood cell count together with prompt improvement of renal function with hydration and transfusion led us to avoid therapeutic plasma exchange. The detection of low levels of vitamin B_12_ and rapid *restitutio ad integrum* with its replacement finally supported the diagnosis. Awareness of clinicians toward this differential diagnosis might spare patients from unnecessary therapeutic plasma exchange that is burdened by a mortality rate of 2.3% and a major complications rate of 24% [16].

## Additional file

Additional file 1: **Figure S1.** Timeline of the diagnostic and therapeutic flow of the present case report. (PDF 101 kb)

## Authors’ information

JV is internal medicine resident at San Gerardo University Hospital, Monza, Italy and University of Milano-Bicocca, Milano, Italy. Her clinical and research interests are focused on hematology and ultrasound imaging, and she has already published an internal medicine case report (Vanoli *et al.*, Medicine 2017). GG is full professor of Internal Medicine at University of Milano-Bicocca, Milano, Italy, and hypertension world expert. MB is assistant professor of Internal Medicine at University of Milano-Bicocca, Milano, Italy.
