# Supplementary material for: Vitamin B12 deficiency-induced pseudothrombotic microangiopathy without macrocytosis presenting with acute renal failure: a case report
Source: J Med Case Rep. 2018 Oct 3;12:296. doi: 10.1186/s13256-018-1815-8 (PMC6169071; doi:10.1186/s13256-018-1815-8)
Supplement: Supplementary file 1 — Figure S1. Timeline of the diagnostic and therapeutic flow of the present case report. (PDF 101 kb) [file 13256_2018_1815_MOESM1_ESM.pdf]

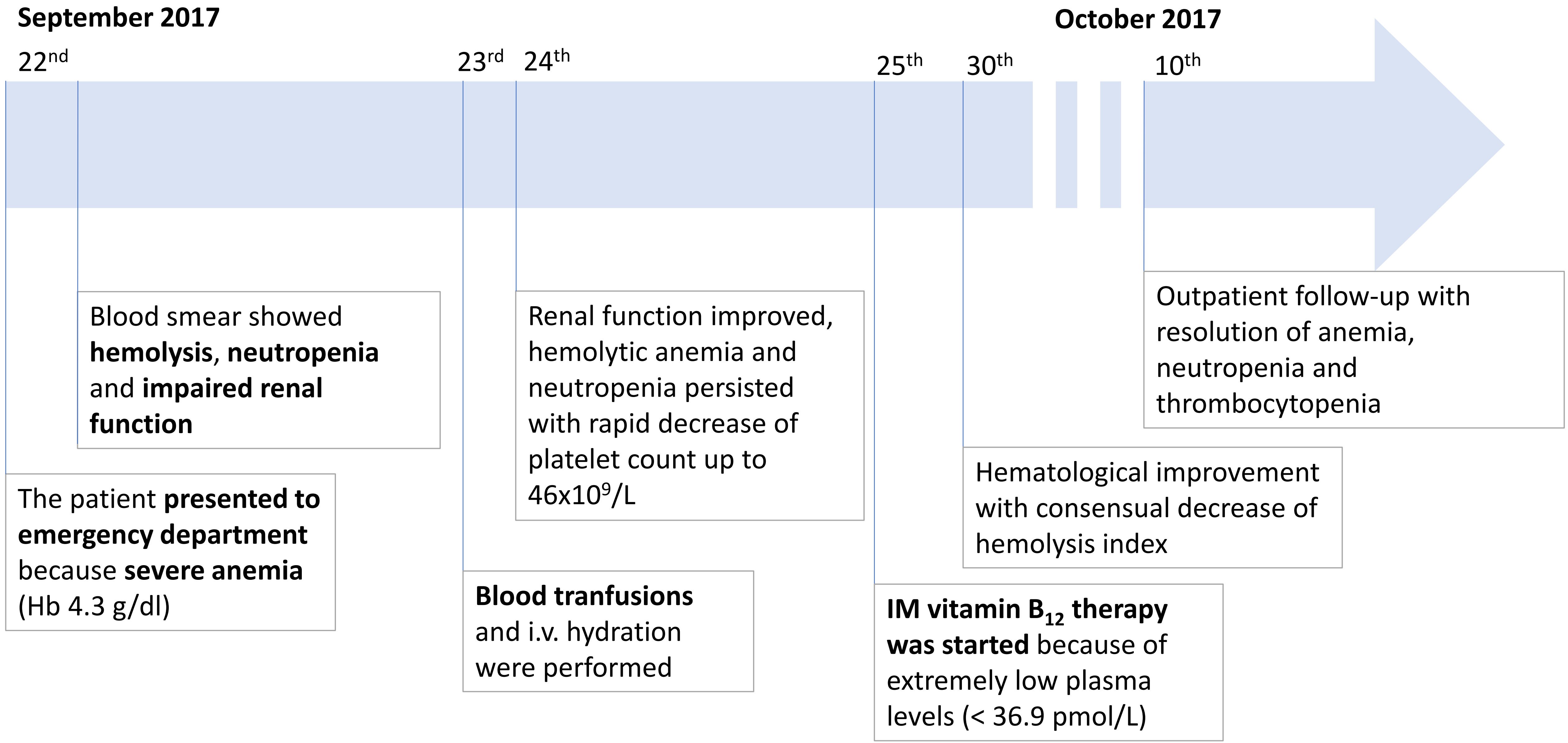
September 2017
22nd
23rd
24th
25th
30th
October 2017
10th
The patient **presented to emergency department** because **severe anemia** (Hb 4.3 g/dl)
Blood smear showed **hemolysis**, **neutropenia** and **impaired renal function**
Blood tranfusions and i.v. hydration were performed
Renal function improved, hemolytic anemia and neutropenia persisted with rapid decrease of platelet count up to $46x10^9$/L
IM vitamin B_{12} therapy was started because of extremely low plasma levels (< 36.9 pmol/L)
Hematological improvement with consensual decrease of hemolysis index
Outpatient follow-up with resolution of anemia, neutropenia and thrombocytopenia
